# Bedaquiline Eliminates Bactericidal Activity of β-Lactams against Mycobacterium abscessus

**DOI:** 10.1128/AAC.00827-19

**Published:** 2019-07-25

**Authors:** Marissa Lindman, Thomas Dick

**Affiliations:** a Center for Discovery and Innovation, Hackensack Meridian Health, Nutley, New Jersey, USA

**Keywords:** *Mycobacterium abscessus*, bedaquiline, β-lactam, drug-drug interaction

## Abstract

The β-lactams imipenem and cefoxitin are used for the treatment of Mycobacterium abscessus lung infections. Here, we show that these cell wall synthesis inhibitors trigger a lethal bacterial ATP burst by increasing oxidative phosphorylation. Cotreatment of M. abscessus with the antimycobacterial ATP synthase inhibitor bedaquiline suppresses this ATP burst and eliminates the bactericidal activity of β-lactams. Thus, the addition of bedaquiline to β-lactam-containing regimes may negatively affect treatment outcome.

## Abstract (precis)

The β-lactams imipenem and cefoxitin are used for the treatment of Mycobacterium abscessus lung infections. Here, we show that these cell wall synthesis inhibitors trigger a lethal bacterial ATP burst by increasing oxidative phosphorylation. Cotreatment of M. abscessus with the antimycobacterial ATP synthase inhibitor bedaquiline suppresses this ATP burst and eliminates the bactericidal activity of β-lactams.

## TEXT

Cell wall synthesis inhibitors such as isoniazid cause a burst in intracellular ATP in Mycobacterium tuberculosis (1, 2). This increase in ATP concentration is suppressed by the ATP synthase inhibitor bedaquiline (BDQ) and protonophore uncouplers of oxidative phosphorylation. This suggests that antimycobacterial cell wall synthesis inhibitors trigger a mycobacterial cell envelope stress response resulting in increased oxidative phosphorylation and, thus, elevated ATP levels (2). Surprisingly, treatment of tubercle bacilli with inhibitors of oxidative phosphorylation suppressed the bactericidal effect of isoniazid, suggesting that the isoniazid-induced ATP burst contributes to cell death mediated by the drug (1, 2).

Lung infections caused by Mycobacterium abscessus, a rapidly growing mycobacterium, are extremely difficult to eradicate (3). Antituberculosis drugs, including isoniazid, are not active against this opportunistic nontuberculous mycobacterial (NTM) pathogen. The macrolides clarithromycin and azithromycin are the main drugs used in combination with parenteral medications (the aminoglycoside amikacin and a β-lactam). The macrolides are the only oral agents reliably active *in vitro* against M. abscessus (4). The β-lactams used for M. abscessus treatment are imipenem and cefoxitin (4). Recent reports suggest that treating with two β-lactam drugs may have benefit (5–7). Furthermore, the new β-lactamase inhibitors relebactam and vaborbactam (8) as well as avibactam (6) were shown to significantly improve *in vitro* activity of various marketed β-lactams (8). These findings suggest β-lactam-containing regimens may be increasingly applied in the future. BDQ, developed as an antituberculosis drug, was shown to be active against M. abscessus (9, 10) and is added in cases of treatment failures (11).

β-Lactams inhibit peptidoglycan synthesis and, thus, interfere with cell wall synthesis. It is not known whether β-lactams, similar to isoniazid in M. tuberculosis, cause an ATP burst in M. abscessus and, if they do, whether this contributes to the bactericidal activity of the drugs. If this is the case, adding BDQ to β-lactam-containing regimens for M. abscessus treatment may be counterproductive.

**FIG 1:**
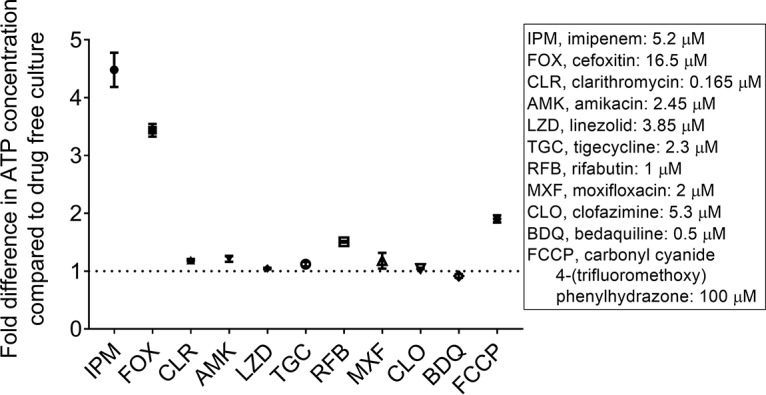
Effect of treatment of M. abscessus cultures with various drugs on bacterial ATP content. Growing cultures were treated for 4 h with inhibitors of peptidoglycan synthesis (imipenem, cefoxitin), inhibitors of protein (clarithromycin, amikacin, linezolid, tigecycline), RNA (rifabutin), DNA synthesis (moxifloxacin), clofazimine, or inhibitors of oxidative phosphorylation—bedaquiline, carbonyl cyanide 4-(trifluoromethoxy)phenylhydrazone—at MICs. ATP content of the cultures was measured and normalized for growth based on each culture’s optical density at 600 nm (OD_600_). On the *y* axis, the fold difference of the ATP level of drug-treated culture compared to the ATP level of drug-free control culture is shown. MIC values are displayed in the right box, together with abbreviation of the drug names. Each experiment was performed at least three times independently. Mean values and standard deviations are shown. Note that whereas non-β-lactam drugs did not affect ATP content of M. abscessus cultures, the protonophore FCCP appears to cause a weak increase under our test conditions. The reason for this effect remains to be determined.

To determine whether β-lactams cause an increase in intrabacterial ATP, exponentially growing cultures of a sequenced clinical strain of M. abscessus (12) were treated with imipenem and cefoxitin for 4 h (about one generation time). ATP contents of cultures were then measured using Promega’s BacTiter-Glo assay as described previously (2). By keeping treatment time short and drug concentrations low (1× MIC), the effects of drug treatment on bacterial viability were minimized. Figure 1 shows that the two β-lactams indeed caused a burst in ATP. To determine whether the ATP increase is specific for these cell wall synthesis inhibitors, we treated M. abscessus cultures with clarithromycin, amikacin, linezolid, tigecycline, rifabutin, moxifloxacin, clofazimine, or bedaquiline. In contrast to the β-lactams, inhibitors of protein, RNA, DNA, or ATP synthesis did not increase ATP levels, suggesting that this effect is specific for agents interfering with cell wall synthesis.

**FIG 2:**
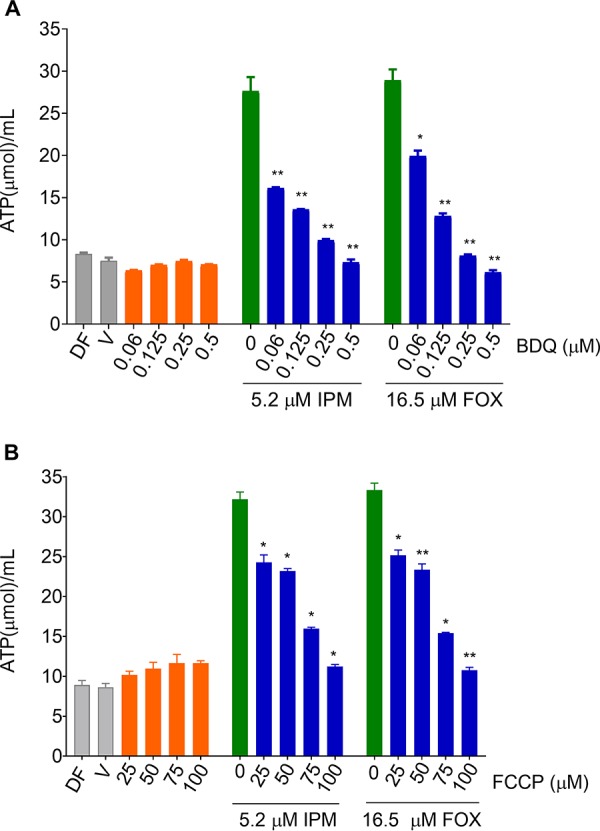
Effect of cotreatment of M. abscessus cultures with β-lactams and inhibitors of oxidative phosphorylation on bacterial ATP content. Growing cultures were treated for 4 h with (A) increasing concentrations of BDQ from 0 μM up to the drug’s MIC (0.5 μM) either without β-lactam added (control, orange bars), together with IPM at MIC (5.2 μM), or with FOX at MIC (16.5 μM). Green bars indicate β-lactam alone. Blue bars indicate β-lactam in combination with increasing BDQ concentrations. (B) Same experiment as in panel A with FCCP replacing BDQ. Cultures were treated with increasing concentrations of FCCP from 0 μM up to the protonophore’s MIC (100 μM) either without β-lactam added, together with IPM at MIC, or with FOX at MIC. For MICs and drug abbreviations, see Fig. 1. The ATP content of cultures after drug treatment was measured and normalized for growth. Each experiment was performed at least three times independently. Asterisks represent the significance determined by the Student’s *t* test comparing the ATP content of the cotreated culture to the ATP content of the β-lactam-only-treated culture as follows: *, <0.05; **; <0.01. ATP content of drug-free (DF) and drug-free plus vehicle (V) cultures. Drug-free indicates that nothing was added to cultures. Vehicle cultures indicate that the same volume of vehicle (dimethyl sulfoxide [DMSO])—without drug—was added that was added to drug cultures.

To determine whether inhibitors of oxidative phosphorylation suppress the ATP burst observed in cultures treated with β-lactams, bacteria were cotreated with a β-lactam and subinhibitory concentrations of either BDQ or an uncoupler of oxidative phosphorylation, the protonophore carbonyl cyanide 4-(trifluoromethoxy)phenylhydrazone (FCCP) (13). Both oxidative phosphorylation inhibitors suppressed the ATP burst triggered by the β-lactams when coadministered (Fig. 2). Together, this suggests that M. abscessus harbors, similar to M. tuberculosis, a cell envelope stress response in which cell wall synthesis inhibitors trigger increased oxidative phosphorylation.

**FIG 3:**
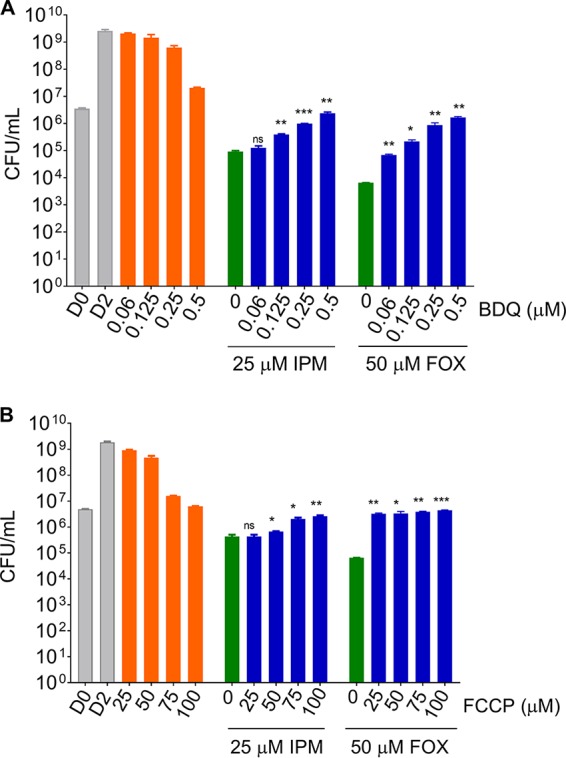
Effect of cotreatment of M. abscessus cultures with β-lactams and inhibitors of oxidative phosphorylation on bacterial viability. Growing cultures were treated for 48 h with (A) increasing concentrations of BDQ from 0 μM up to the drug’s MIC (0.5 μM) either without β-lactam added (control, orange bars), together with IPM at 5× MIC (25 μM), or with FOX at 3× MIC (50 μM). Green bars indicate β-lactam alone. Blue bars indicate β-lactam in combination with increasing BDQ concentrations. (B) Same experiment as in panel A with FCCP replacing BDQ. Cultures were treated with increasing concentrations of FCCP from 0 μM up to the protonophore’s MIC (100 μM) either without β-lactam added, together with IPM at 5× MIC, or with FOX at 3× MIC. For MICs and drug abbreviations, see Fig. 1. Viability of cultures was measured by CFU enumeration on agar. Each experiment was performed at least three times independently. Asterisks represent the significance determined by the Student’s *t* test comparing the recovered bacteria from the cotreated culture to the bacteria recovered from the β-lactam-only-treated culture as follows: *, <0.05; **, <0.01; ***, <0.001. D0 and D2, CFU of drug-free control cultures at the beginning and end of the experiment.

To determine whether suppression of the ATP burst by BDQ affects the bactericidal activity of β-lactams, the effect of β-lactam–BDQ cotreatment on the viability of M. abscessus cultures was determined. To achieve substantial killing by the β-lactams, the treatment time was extended from 4 to 48 h and the concentrations were increased from 1× to 3 to 5× MIC (which is still in the pharmacologically achievable range [14, 15]). Figure 3 shows that the β-lactams alone exerted bactericidal activity. Addition of BDQ to β-lactam-containing cultures reduced the bactericidal activity of the β-lactams in a dose-dependent manner (Fig. 3A). The same effect was observed for cotreatment of cultures with the protonophore FCCP (Fig. 3B). These results suggest that suppression of the β-lactam-induced ATP burst reduces the bactericidal effect of these drugs.

We show here that BDQ antagonizes the bactericidal activity of β-lactams in M. abscessus. Drug-drug potency interaction studies *in vitro* are typically carried out using growth assays, thus addressing the question of whether drugs antagonize in terms of growth inhibition as opposed to a bactericidal effect. To determine whether the observed antagonistic effect of BDQ on the bactericidal activity of β-lactams extends to growth inhibition, we carried out checkerboard analyses and measured turbidity as a readout for growth as described (16, 17) (see Fig. S1 in the supplemental material). These experiments revealed that BDQ–β-lactam combinations are additive, i.e., not antagonistic, in growth inhibition assays. Thus, there is a disconnect between drug-drug potency interaction regarding growth inhibition and bactericidal activity, and the antagonistic effect of BDQ on the bactericidal activity of β-lactams would have been missed if only standard checkerboard analyses had been carried out.

In conclusion, we show that imipenem and cefoxitin trigger an ATP burst in M. abscessus by increasing oxidative phosphorylation. Treatment of M. abscessus with BDQ or a protonophore suppressed the ATP burst and, importantly, eliminated the bactericidal activity of the β-lactams. Imipenem or cefoxitin are often used in regimens to treat lung disease caused by this NTM pathogen (3). Our *in vitro* data suggest that the addition of BDQ to β-lactam-containing regimens may negatively affect treatment outcome. Murine studies are warranted to test for antagonism of β-lactam–bedaquiline combinations *in vivo* to provide evidence that our finding may be of clinical relevance.

Our results suggest that M. abscessus, similar to M. tuberculosis (2), harbors a cell envelope stress response in which inhibition of cell wall synthesis triggers increased oxidative phosphorylation resulting in a burst of ATP. This ATP surge appears to contribute to the bactericidal activity across different classes of antimycobacterial cell wall synthesis inhibitors, including isoniazid in the case of M. tuberculosis and β-lactams in the case of M. abscessus. It is interesting to note that our findings support a new concept in understanding antibiotic death physiology suggested recently by Collins and colleagues. The authors propose that drug-induced changes in ATP homeostasis critically drive lethal metabolic alterations (18).

Our study focused on the interaction of β-lactams and BDQ in M. abscessus, not M. tuberculosis. It is important to note that β-lactams (meropenem or imipenem-cilastatin along with amoxicillin-clavulanate) are used for the treatment of multidrug-resistant (MDR) and extensively drug-resistant (XDR) tuberculosis, often together with BDQ (19). Furthermore, BDQ is increasingly considered in novel regimens for the treatment of drug-susceptible tuberculosis (20). Whether BDQ also antagonizes the activity of β-lactams in M. tuberculosis has not been determined. However, it was shown that meropenem (as well as the two mechanistically different cell wall inhibitors, ethambutol and the benzothiazinone BTZ043) triggered an ATP burst in tubercle bacilli similar to isoniazid (1). Considering the clinical practice of coadministering β-lactams and BDQ, an antagonistic effect would have far-reaching implications for therapy of drug-resistant tuberculosis. Thus, *in vitro* and *in vivo* studies are urgently required to address a possible antagonism of β-lactams and BDQ in M. tuberculosis.

## Supplementary Material

Supplemental file 1
