# Supplementary material for: Bedaquiline Eliminates Bactericidal Activity of β-Lactams against Mycobacterium abscessus
Source: Antimicrob Agents Chemother. 2019 Jul 25;63(8):e00827-19. doi: 10.1128/AAC.00827-19 (PMC6658768; doi:10.1128/AAC.00827-19)
Supplement: Supplemental file 1 [file AAC.00827-19-s0001.pdf]

Supplemental materials

Bedaquiline eliminates bactericidal activity of β-lactams against *Mycobacterium abscessus*

Marissa Lindman and Thomas Dick

Supplemental Figure 1

	MIC				FICI
	β-lactam		Bedaquiline		
	Alone	Combined	Alone	Combined	
Imipenem	6.25	0.78	0.39	0.195	0.625
Cefoxitin	25	6.25	0.39	0.195	0.75

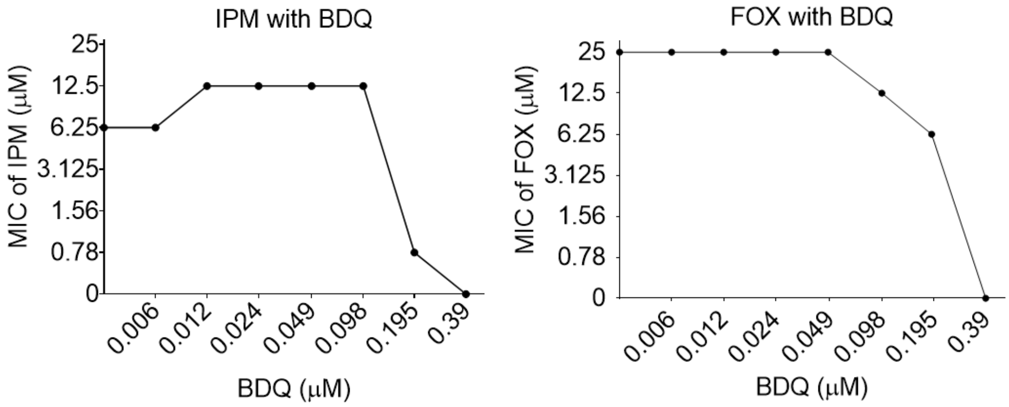

Figure S1 Effect of combination of β-lactams and bedaquiline on growth of *M. abscessus*. Results of checker board analyses are shown. The checker board assay was carried out as described previously (1, 2). Synergy is defined as FICI ≤ 0.5, indifference is defined as 0.5 < FICI ≤ 4, and antagonism is defined as FICI > 4 (2). For drug abbreviations see Figure 1. The experiment was carried out twice and a representative example is shown.

REFERENCES

1. Aziz DB, Teo JWP, Dartois V, Dick T. 2018. Teicoplanin – Tigecycline Combination Shows Synergy Against Mycobacterium abscessus. Frontiers in Microbiology 9.
2. Hsieh MH, Yu CM, Yu VL, Chow JW. 1993. Synergy assessed by checkerboard a critical analysis. Diagnostic Microbiology and Infectious Disease 16:343-349.
